# Multiple endocrine neoplasia type 1 with concurrent Cushing’s disease, prolactinoma, and multifocal pancreatic neuroendocrine tumors including insulinoma: a case report

**DOI:** 10.48101/ujms.v131.14421

**Published:** 2026-08-06

**Authors:** Beatriz Tavares da Silva, Diogo Brandão Neves, Maria Teresa Pereira, Carolina Noronha, Isabel Ribeiro, Cláudia Amaral

**Affiliations:** a Clinical Outpatient of Endocrinology, Unidade Local de Saúde de Santo António, Porto, Portugal; b Clinical Outpatient of Neurosurgery, Unidade Local de Saúde de Santo António, Porto, Portugal

**Keywords:** Multiple endocrine neoplasia type 1, Cushing’s disease, insulinoma, diagnostic imaging

## Abstract

**Background:**

Multiple Endocrine Neoplasia type 1 *(MEN1)* is an autosomal dominant tumor predisposition syndrome involving primary hyperparathyroidism (PHPT), pituitary neuroendocrine tumors (PitNETs), and pancreatic neuroendocrine tumors. MEN1-associated insulinomas may present with multifocal disease, complicating management, while Cushing’s disease is uncommon, and the coexistence of multiple functioning tumors complicates management.

**Case presentation:**

A 28-year-old man presented with adrenocorticotropic hormone-dependent hypercortisolism and progressive cushingoid features. Pituitary Magnetic Resonance Imaging revealed a microadenoma, confirmed as a PitNET on histopathology. Concurrent PHPT and genetically confirmed MEN1 (splice-site pathogenic variant c.825-1G>A) were identified. Transsphenoidal surgery failed to achieve remission, and metyrapone provided partial control. Subtotal parathyroidectomy resulted in persistent PHPT. Imaging revealed multifocal pancreatic NETs with biochemically confirmed insulinoma. Lanreotide achieved complete resolution of hypoglycemia, while ^68^Ga-NOTA-exendin-4 positron emission tomography/computed tomography did not identify a dominant lesion, supporting conservative management.

**Conclusion:**

This case highlights the complexity of MEN1 with multiple functioning tumors and the clinical impact of hormonal interactions. It underscores the importance of multidisciplinary, individualized management, particularly when tumor multifocality limits surgical options. It also emphasizes the need to recognize complex endocrine syndromes in general clinical practice.

## Introduction

Multiple Endocrine Neoplasia type 1 (*MEN1*) is a rare autosomal dominant syndrome caused by germline inactivating pathogenic variants in the *MEN1* gene, which encodes menin, a tumor suppressor involved in transcriptional regulation and genome stability (1, 2). *MEN1* predisposes to multiple endocrine tumors, classically affecting the parathyroid glands, pancreatic neuroendocrine tumors (pNETs), and pituitary neuroendocrine tumors (PitNETs) (1, 3). Primary hyperparathyroidism (PHPT) is the most frequent and often earliest manifestation. MEN1-associated PHPT is typically more severe than sporadic disease, with earlier onset and greater risk of renal and skeletal complications (1, 3, 4).

pNETs are observed in up to 70–80% of MEN1 patients and represent a major source of morbidity (5). While insulinomas are usually solitary, multifocal pancreatic involvement may occur in MEN1 and can complicate tumor localization (1, 5, 6). Pituitary involvement is commonly lactotroph-predominant; conversely, corticotroph PitNETs causing Cushing’s disease (CD) are uncommon (less than 5%), and the coexistence of distinct functional PitNETs is particularly rare (less than 1%) (7, 8). *MEN1*-associated PitNETs may also show more aggressive behavior, although current evidence suggests that treatment response of *MEN1*-associated PitNETs is not significantly different from sporadic cases (9).

We report a young man with *MEN1* presenting with CD, hyperprolactinemia consistent with lactotroph involvement, PHPT, and multifocal pNETs including a biochemically confirmed insulinoma. The case highlights diagnostic ambiguity driven by tumor multifocality and overlapping endocrine syndromes, and the need for multidisciplinary, individualized management.

## Case report

### Initial presentation and endocrine evaluation

A 28-year-old previously healthy man presented with progressive cushingoid features, including a 30-kg weight gain (approximately 23% from baseline) and central obesity (body mass index 40.4 kg/m^2^), followed by the development of violaceous abdominal striae and facial fullness. Physical examination also revealed several large subcutaneous lipomas. Blood pressure at presentation was 142/73 mmHg. Initial laboratory evaluation demonstrated hypercalcemia (albumin-corrected calcium of 12.3 mg/dL, reference: 8.6–10.2), hypophosphatemia (2.1 mg/dL, reference: 2.5–4.5), and markedly elevated parathyroid hormone (PTH 270.4 pg/mL, reference: 15–65), consistent with PHPT. In parallel, CD was suspected based on persistently elevated 24-h urinary free cortisol (UFC) – 181.0 µg/24 h and 198.0 µg/24 h (reference: 5.2–136). This was accompanied by high-normal plasma adrenocorticotropic hormone (ACTH) levels (47.2 pg/mL, reference: 10–60). Family history was negative for *MEN1*-related tumors.

The patient was referred to the Endocrinology Department in April 2024 for comprehensive evaluation of these findings. The clinical timeline, summarizing the onset of symptoms and the sequential multidisciplinary interventions, is detailed in Table 1. Table 2 provides the key biochemical findings before and after the performed treatments.

**Table 1:** Clinical timeline of a patient with MEN1, summarizing the onset of symptoms, key hormonal investigations, pituitary and pancreatic imaging studies, genetic confirmation, and sequential treatments for Cushing’s disease, primary hyperparathyroidism, and pancreatic neuroendocrine tumors including insulinoma.

Date (month/year)	Clinical domain	Key findings and interventions
April 2024	Initial presentation	Referral to Endocrinology due to progressive cushingoid features, hypercalcemia and suspected ACTH-dependent hypercortisolism
May–June 2024	CD	Biochemical confirmation of ACTH-dependent hypercortisolism (elevated midnight salivary cortisol and UFC); pituitary MRI revealing a 7-mm microadenoma; inferior petrosal sinus sampling confirming pituitary source
June 2024	PHPT	Severe hypercalcemia requiring intravenous zoledronic acid; imaging suggesting multiglandular parathyroid disease
July 2024	Medical therapy (pituitary)	Initiation of cabergoline for hyperprolactinemia and CD
September 2024	Pituitary surgery	Transsphenoidal resection for CD; histopathology showing a plurihormonal PitNET (PRL+/GH+, ACTH-)
October 2024	Postoperative assessment	Persistent hypercortisolism; early postoperative pituitary MRI demonstrating residual sellar lesion (~7 mm) abutting the cavernous sinus
October–November 2024	Pancreatic NET work-up	^68^Ga-DOTATATE PET/CT and endoscopic ultrasound identifying multiple pancreatic neuroendocrine tumors; biopsy confirming well- differentiated pNET (Ki-67 <3%)
November 2024	Medical therapy (CD)	Initiation of metyrapone for persistent hypercortisolism with subsequent biochemical and clinical improvement
November 2024	Insulinoma	Supervised fasting test confirming endogenous hyperinsulinism; recurrent hypoglycemia after metyrapone; initiation of lanreotide with resolution of hypoglycemic episodes
December 2024	Insulinoma localization	^68^Ga-NOTA-exendin-4 PET/CT performed for insulinoma localization; no dominant GLP-1R-avid lesion identified
January–June 2025	Follow-up	Ongoing medical management of hypercortisolism, insulinoma and PHPT; continuous glucose monitoring showing no further hypoglycemia
July 2025	Parathyroid surgery	Subtotal parathyroidectomy with histology consistent with parathyroid hyperplasia; persistent biochemical hyperparathyroidism postoperatively
August 2025	Lipoma	Surgical excision of large, symptomatic subcutaneous abdominal lipomas
November 2025–January 2026	Persistent PHPT and persistent CD	SPECT/CT identifying residual hyperfunctioning parathyroid tissue; consideration of re- intervention if medical therapy fails/not tolerable, and patient awaiting repeat pituitary MRI for planning of potential repeat surgery

ACTH: adrenocorticotropic hormone; CD: Cushing’s disease; CT: computed tomography; GH: growth hormone; GLP-1: glucagon-like peptide-1; Ki-67: proliferation index Ki-67; MRI: magnetic resonance imaging; PET/CT: positron emission tomography/computed tomography; PHPT: primary hyperparathyroidism; PitNET: pituitary neuroendocrine tumor; pNET: pancreatic neuroendocrine tumor; PRL: prolactin; SPECT/CT: single-photon emission computed tomography/computed tomography; UFC: urinary free cortisol.

**Table 2:** Biochemical profile before and after therapeutic interventions.

Parameter (units)	Pre-treatment	Post-treatment	Reference range
Albumin-corrected calcium (mg/dL)	12.3	10.7	8.6–10.2
Phosphorus (mg/dL)	2.1	2.5	2.5–4.5
Parathyroid hormone (pg/mL)	270.4	246.3	15–65
24-h urinary free cortisol (µg/24h)	181; 198	61.6	5.2–136
Midnight salivary cortisol (µg/dL)	0.478	0.321	< 0.261
ACTH (pg/mL)	47.2	43.4	10–60
Prolactin (ng/mL)	65.6	0.8	4.04–15.20
Fasting glucose (mg/dL)	47	81	55–100
Insulin (µU/mL)	33	Not assessed	<10
C-peptide (ng/mL)	7.28	Not assessed	0.5–2.0

ACTH: adrenocorticotropic hormone.

### ACTH-dependent hypercortisolism (CD)

Biochemical reassessment confirmed ACTH-dependent Cushing’s syndrome, characterized by elevated midnight salivary cortisol (0.478 µg/dL; reference: <0.261), increased 24-h UFC (187.0 µg/24h; reference: 4.3–176.0), and persistently elevated ACTH (40.8 pg/mL, reference: 10–60). Additional endocrine evaluation revealed multiaxis pituitary involvement, including hyperprolactinemia (65.6 ng/mL; reference: 4.04–15.20), hypogonadotropic hypogonadism (FSH 4.6 mIU/mL, LH 3.5 mIU/mL, and total testosterone 0.80 ng/mL [reference: 2.8–8.0]), and central hypothyroidism (FT4 0.95 ng/dL, reference: 1.01–1.65; TSH 3.07 µUI/mL, reference: 0.30–3.18).

Pituitary Magnetic Resonance Imaging (MRI) identified a 7-mm right-sided pituitary microadenoma. Inferior petrosal sinus sampling (IPSS) demonstrated a central-to-peripheral ACTH gradient of 16 at baseline and 75 after stimulation, supporting a pituitary source of ACTH secretion. However, due to technical inability to catheterize the left inferior petrosal sinus, lateralization could not be reliably assessed. The patient underwent transsphenoidal pituitary surgery in September 2024. Histopathological examination revealed a plurihormonal PitNET. Immunohistochemical analysis demonstrated strong prolactin expression with focal growth hormone positivity and absence of ACTH staining. The Ki-67 proliferation index was low (< 3%), consistent with a well-differentiated tumor. Transcription factor analysis (PIT1, TPIT, SF1) was not available. Immediate postoperative morning serum cortisol remained elevated (18.5 µg/dL), indicating a lack of biochemical remission.

Postoperative pituitary MRI demonstrated a residual 6-mm lobulated lesion in the left sellar region with slight cavernous sinus extension. Given persistent hypercortisolism (midnight salivary cortisol 0.478 µg/dL, reference: < 0.261), medical therapy with cabergoline (1.5 mg/week) and metyrapone (750 mg/day) was initiated. Prolactin levels normalized postoperatively (0.8 ng/mL), prior to initiation of dopamine agonist therapy. While this strategy initially achieved clinical improvement and a reduction in cortisol (UFC 61.6 µg/24 h, reference: 4.3–176.0), only partial biochemical control was sustained, requiring a dose escalation of metyrapone to 1,000 mg/day. The patient is currently awaiting reassessment for potential surgical reintervention.

### Primary hyperparathyroidism

Repeat evaluation confirmed persistent PHPT, with hypercalcemia (12.7 mg/dL, reference: 8.6–10.2 mg/dL), hypophosphatemia (2.23 mg/dL, reference: 2.5–4.5 mg/dL), and elevated PTH (229 pg/mL, reference: 15–65 pg/mL). Parathyroid scintigraphy demonstrated bilateral hyperfunctioning tissue, and renal ultrasound revealed a 7-mm calculus. Bone densitometry showed osteopenia (*Z*-score –1.8 at the wrist; –1.0 at the lumbar spine and femoral neck). Intravenous zoledronic acid was administered for metabolic stabilization.

In July 2025, the patient underwent subtotal parathyroidectomy (3^⅓^ glands) combined with transcervical thymectomy. Histopathological examination confirmed diffuse parathyroid hyperplasia. However, postoperative biochemical assessment demonstrated persistent PHPT (PTH 221.0 pg/mL; albumin-corrected calcium 11.2 mg/dL; phosphorus 1.7 mg/dL). Medical therapy with denosumab was initiated. Although cinacalcet was initially discontinued due to gastrointestinal intolerance, an alternate-day dosing regimen was subsequently introduced to improve tolerability. This strategy resulted in partial biochemical improvement, with albumin-corrected calcium of 10.7 mg/dL (reference: 8.6–10.2 mg/dL), phosphatemia of 2.5 mg/dL (reference: 2.5–4.5 mg/dL), and persistently elevated PTH levels (246.3 pg/mL; reference: 15–65 pg/mL). If this conservative approach fails to achieve sustained biochemical control or remains poorly tolerated, surgical re-exploration with resection of half of the remaining parathyroid remnant is planned.

### MEN1 diagnosis and pNETs including insulinoma

**Figure 1:**
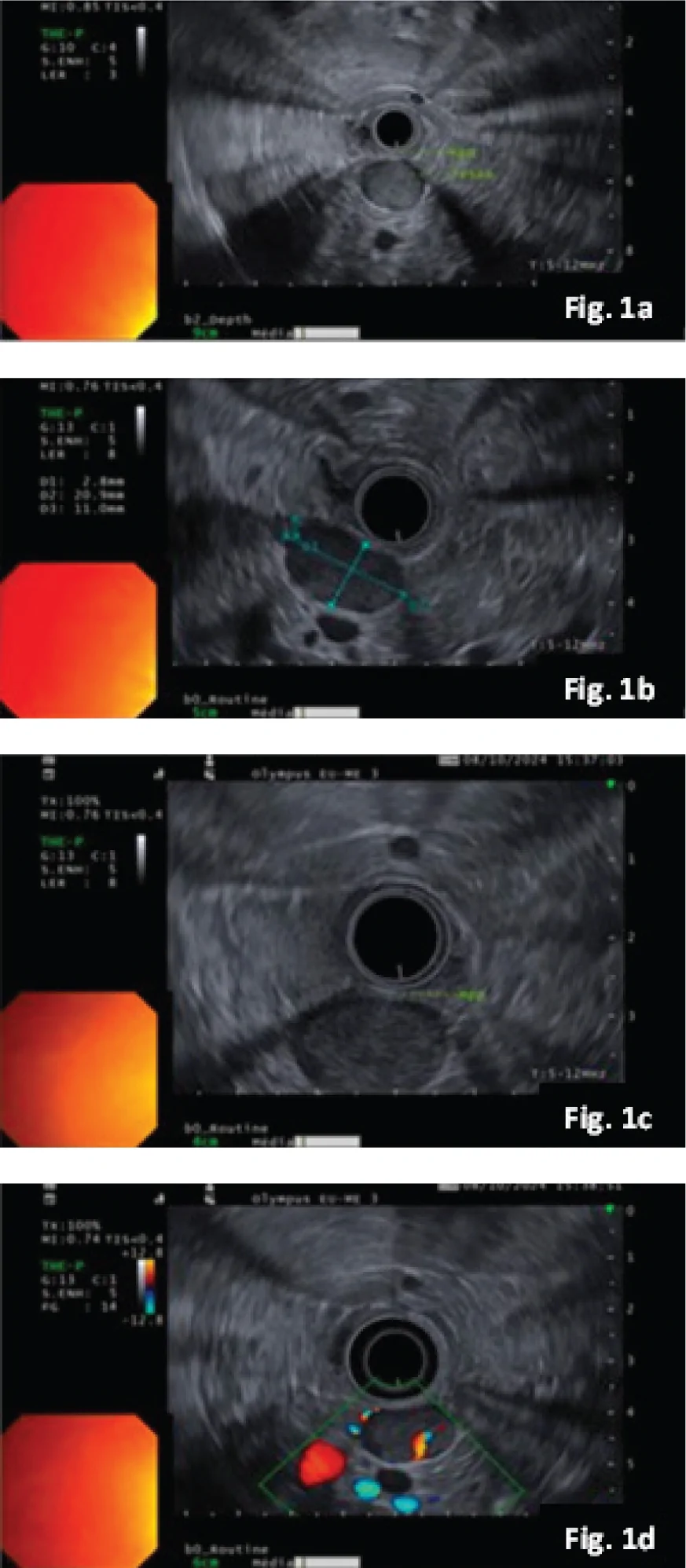
Endoscopic ultrasound (EUS) of the pancreas. (a–d) Linear-array EUS images demonstrating multiple well-demarcated, hypoechoic, solid pancreatic nodules in the body and tail, consistent with multifocal pancreatic neuroendocrine tumors. Surrounding vascular structures were preserved. Fine-needle aspiration confirmed a well-differentiated neuroendocrine tumor with a Ki-67 index < 3%.

A pathogenic MEN1 splice-site variant (c.825-1G>A) was identified, confirming the diagnosis of *MEN1*. Abdominal CT initially revealed two hypervascular nodules in the pancreatic tail (1.4 and 1.6 cm), suspicious for pNETs. Subsequent ^68^Ga-DOTATATE positron emission tomography/computed tomography (PET/CT) demonstrated four somatostatin receptor-positive pancreatic lesions, and endoscopic ultrasound further identified four well-demarcated hypoechoic pancreatic nodules, including a 20.9 × 11 mm lesion adjacent to the uncinate process (Figure 1a–d). Fine-needle aspiration cytology confirmed a well-differentiated neuroendocrine tumor with a Ki-67 index < 3%.

**Figure 2:**
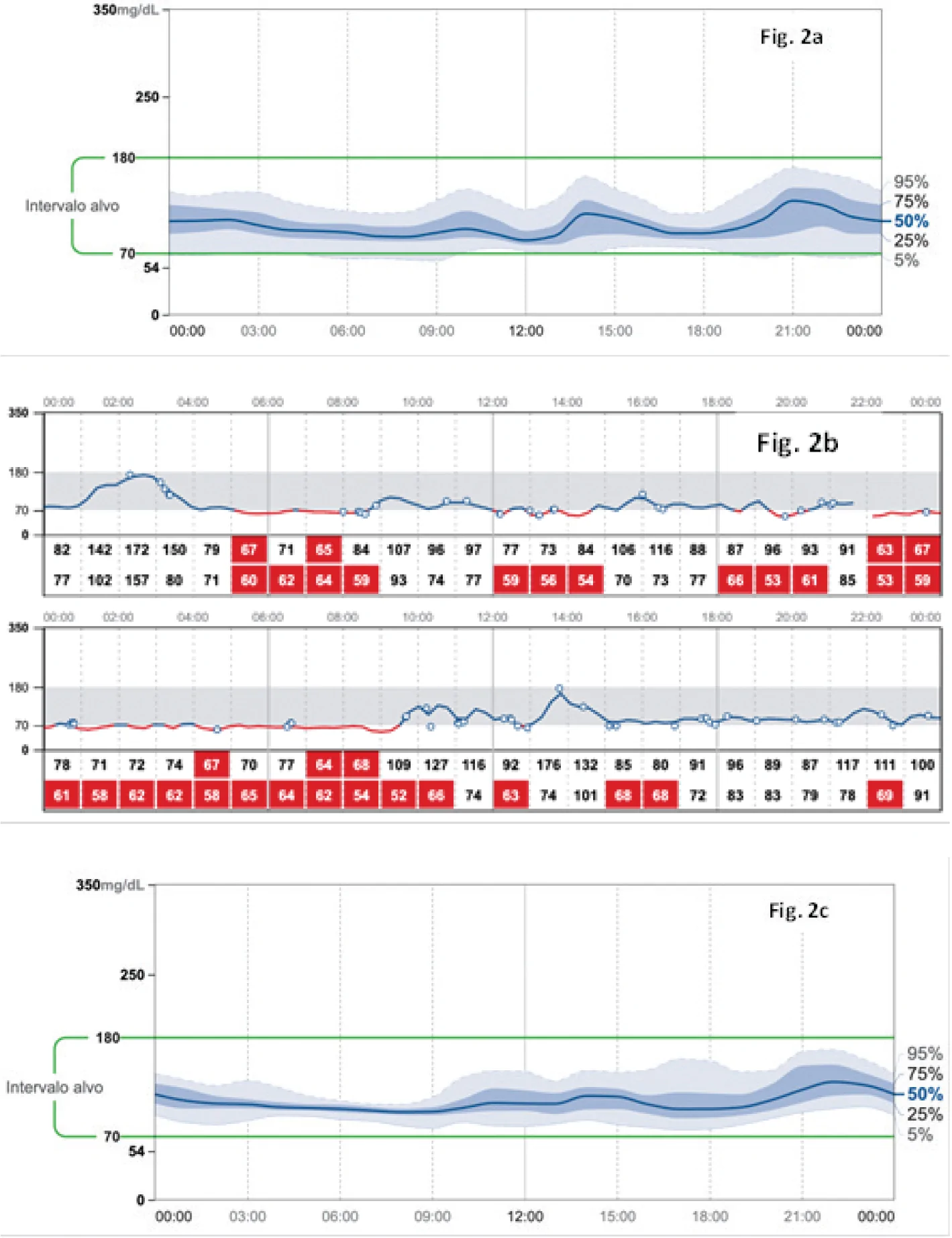
Continuous glucose monitoring (CGM) before and after initiation of lanreotide. (a) Ambulatory Glucose Profile (AGP) prior to treatment demonstrating marked glycemic instability and recurrent hypoglycemic episodes, particularly during nocturnal and fasting periods. (b) Representative daily glucose tracings (October 2024) illustrating high frequency of clinically significant hypoglycemia. (c) Post-treatment AGP following lanreotide initiation, showing stabilized glucose variability and near-complete suppression of hypoglycemic events, with the majority of values within the target range (70–180 mg/dL).

The diagnosis of a functioning insulinoma emerged during the preoperative period for pituitary surgery. The prolonged fasting required for the management of CD unmasked a state of severe hypoglycemia, initially noted on routine blood work with a plasma glucose of 42 mg/dL. The patient reported recurrent nocturnal and fasting-related hypoglycemic symptoms, including diaphoresis, tremor, fatigue, and intermittent confusion compatible with neuroglycopenia. A formal 12-h fast, terminated early due to symptomatic hypoglycemia fulfilling Whipple’s triad, confirmed endogenous hyperinsulinism (glucose 47 mg/dL, insulin 33 µU/mL [reference: < 10], and C-peptide 7.28 ng/mL [reference: 0.5–2.0]), fulfilling the diagnostic criteria for insulinoma. The severity of these hypoglycemic episodes was objectively captured by continuous glucose monitoring (CGM), which demonstrated frequent and clinically significant nocturnal events (Figure 2a–b).

**Figure 3:**
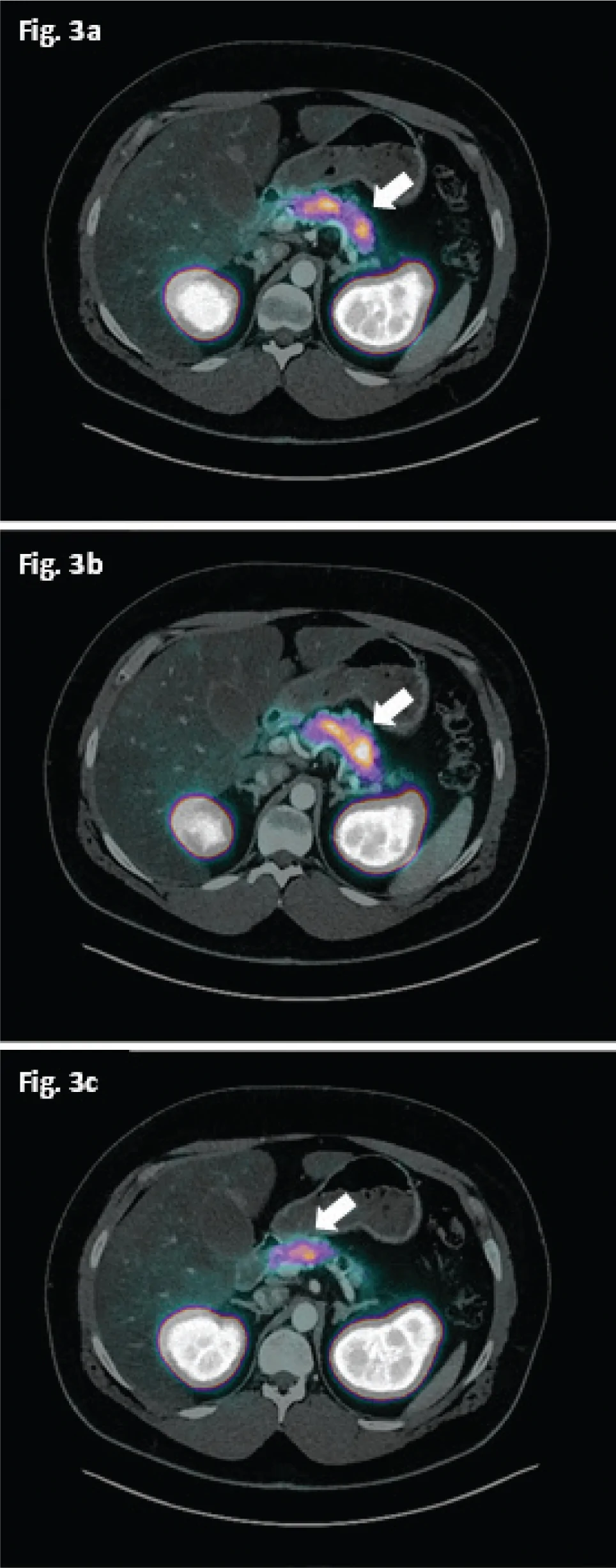
^68^Ga-NOTA-exendin-4 PET/CT images showing GLP-1 receptor uptake in the pancreatic region in the context of MEN1-associated multifocal pancreatic neuroendocrine tumors. Consecutive axial fused PET/CT images demonstrate uptake corresponding to the pancreatic lesions, indicated by arrows. No single dominant GLP-1R-avid lesion was identified to account for the patient’s hyperinsulinemic hypoglycemia, limiting surgical localization. PET/CT: positron emission tomography/computed tomography; GLP-1: glucagon-like peptide-1; MEN1: Multiple Endocrine Neoplasia type 1.

Following discussion at a multidisciplinary endocrine tumor board, medical therapy with lanreotide autogel (120 mg every 28 days) was initiated. This served a dual purpose: providing symptomatic treatment for the insulinoma and utilizing its antiproliferative effects for the management of the multiple pNETs. After the initiation of lanreotide, the ambulatory glucose profile showed near-complete suppression of hypoglycemic events (Figure 2c). This biochemical improvement was accompanied by complete resolution of hypoglycemic symptoms. To support potential surgical planning, advanced localization with ^68^Ga-NOTA-exendin-4 PET/CT was performed; however, no single dominant insulinoma focus was identified (Figure 3). This conservative pharmacological approach led to marked clinical improvement, with complete resolution of hypoglycemic episodes and documented disease stability on the most recent abdominal MRI in January 2026.

### Follow-up and ongoing management

At the most recent follow-up in January 2026, the patient remained clinically stable with no recurrence of hypoglycemic episodes, although persistent cushingoid features were still noted. During this period, the patient also underwent surgical excision of large, symptomatic subcutaneous lipomas. No evidence of bronchopulmonary, thymic, duodenal neuroendocrine tumors or adrenal lesions was identified during the evaluation. The current therapeutic regimen consists of medical therapy with lanreotide, cabergoline, and metyrapone, supplemented by endocrine replacement and antihypertensive treatment. Ongoing surveillance is being maintained through serial biochemical testing and cross-sectional imaging, while further surgical strategies for both pituitary and parathyroid disease remain under multidisciplinary evaluation.

## Discussion

This case highlights the diagnostic and therapeutic challenges of *MEN1* when multiple functioning tumors coexist. Although the classic *MEN1* triad is well recognized, phenotypic heterogeneity often complicates the clinical course. In this patient, diagnostic suspicion was reinforced by non-endocrine manifestations, particularly subcutaneous lipomas, a common cutaneous feature of the syndrome. These findings emphasize the importance of maintaining a high index of suspicion in patients with pluriglandular involvement.

Pituitary disease in *MEN1* is typically lactotroph-predominant, whereas CD is rare (7, 8). This case is notable for CD confirmed by IPSS, associated with hyperprolactinemia and a plurihormonal PitNET phenotype. Although ACTH immunostaining was negative and postoperative remission was not achieved, the diagnosis of pituitary ACTH-dependent disease was supported by IPSS demonstrating a significant central-to-peripheral ACTH gradient. Ectopic ACTH secretion was considered in the differential diagnosis given the presence of multiple neuroendocrine tumors; however, imaging studies did not identify a thoracic or extrapituitary ACTH-secreting lesion. Plurihormonal PitNETs have been described in *MEN1* and may reflect lineage plasticity or technical limitations of immunohistochemistry (4, 8). MEN1-associated PitNETs may also behave more aggressively, contributing to persistent disease after surgery (9). Repeat surgery was deferred due to cavernous sinus invasion, which is associated with lower remission rates and higher morbidity, particularly in *MEN1*-related tumors (9). Reoperation also carries increased risk of hypopituitarism, vascular injury, and cranial nerve deficits (10). Medical therapy was therefore used as a bridge to re-intervention, with metyrapone providing biochemical control and agents such as osilodrostat or relacorilant representing additional options (10, 11). Pasireotide could also be considered given its activity in corticotroph and neuroendocrine tumors (12, 13); however, its off-label use limited its applicability in this case.

PHPT is usually the earliest and most penetrant *MEN1* manifestation and frequently requires surgery due to multiglandular involvement (1, 3, 14). In this patient, severe biochemical disease with renal and skeletal complications supported early surgical intervention.

Management of multifocal pNETs remains challenging, particularly when insulinoma coexists with other lesions (5, 6, 15, 16). In this case, the interaction between hormonal excess states was clinically relevant. Hypercortisolism may have attenuated hypoglycemia through cortisol-induced insulin resistance (1, 6). Hypoglycemia became evident only after treatment of CD, highlighting this physiological antagonism and its potential clinical impact (6, 16). Multifocality and small lesion size limit the sensitivity of conventional and functional imaging (5, 6). Although somatostatin receptor PET/CT (17) and glucagon-like peptide-1 (GLP-1) receptor imaging (18, 19) improve detection, they may fail to identify a dominant lesion. Even after extensive evaluation, localization may remain inconclusive (14, 16, 17). SACST is recommended in such settings (6, 20), but in this case its utility was limited by the presence of multiple lesions within the same pancreatic regions and the low likelihood of altering surgical strategy. Initial suspicion of lymph node involvement was not confirmed after multidisciplinary review (6, 15).

Surgical management of MEN1-associated pNETs is further complicated by the need to balance disease control and preservation of pancreatic function. While extensive pancreatic resection may achieve biochemical control, it is associated with significant long-term morbidity (21, 22). Importantly, the largest pancreatic lesion exceeded 2 cm, a threshold generally associated with increased metastatic potential in MEN1-associated pNETs and frequently considered an indication for surgical intervention (15, 21). However, the absence of a clearly dominant insulin-secreting lesion, the multifocal pancreatic disease burden, and the favorable biochemical and clinical response to lanreotide supported an initial conservative multidisciplinary approach.

Although multiple functioning tumors are well recognized in MEN1, the coexistence of CD and insulinoma is rarely reported. Most cases describe isolated pituitary or pancreatic involvement, or combinations of non-functioning tumors, highlighting the heterogeneity of the syndrome (23–25). Insulinomas are often multifocal and may recur, further complicating management (23, 25). This case underscores the need for individualized, multidisciplinary decision-making, taking into account tumor dominance, hormonal activity, and feasibility of intervention.

## Conclusion

This case underscores the need for individualized, multidisciplinary decision-making, particularly in the presence of interacting hormonal excess states. Early genetic diagnosis, coupled with advanced functional imaging and strategic therapeutic sequencing, remains pivotal in optimizing outcomes. Management of *MEN1* requires careful sequencing of interventions, balancing competing endocrine risks, specifically the dangerous interplay between hypercortisolism and hypoglycemia, while addressing multiglandular and multifocal disease.

## Data Availability

The data that support the findings of this study are not publicly available due to ethical and legal restrictions related to patient confidentiality. It may be made available upon reasonable request and institutional approval.
